# Use of Focus Group as Selection Method of Descriptors for Check-All-That-Apply (CATA) for Sensory Characteristics of Hot Dogs

**DOI:** 10.3390/foods11030269

**Published:** 2022-01-20

**Authors:** Isabela Rodrigues, Danielle Rodrigues Magalhaes, Marco Antonio Trindade

**Affiliations:** Department of Food Engineering, University of Sao Paulo, Pirassununga 13635-900, SP, Brazil; isabelarodrigues@alumni.usp.br (I.R.); d.magalhaes@usp.br (D.R.M.)

**Keywords:** CATA, sodium reduction, meat products, sensory analysis, consumer perception

## Abstract

Check-all-that-apply (CATA) is a methodology for sensory product characterization that can be used by consumers. These characteristics, on the other hand, are determined by a trained panel, and consumers are not asked how they perceive these attributes; as a result, some of the characteristics raised by the trained panel may not be relevant to consumers. In this study, the CATA test was applied to characterize three types of hot dogs, those with or without irradiation and salt reduction, and the focus group (FG) technique was employed to determine the CATA descriptors. Each participant in the FG provided five words (attributes) that, in their opinion, best defined each sample. Then, to understand the meaning and to assemble each of the different terms, a discussion of the defined attributes was conducted. The list of CATA descriptors was compiled using the most often cited attributes. The findings indicate that the major difference in hot dogs was between the formulations with and without sodium reduction. The consumers only noticed minor effects resulting from the irradiation process. The use of focus group as the method to select the CATA descriptors related to hot dogs was proven to be valid since the words that were listed for these samples were attributes that typically characterize hot dog sausages.

## 1. Introduction

Check-all-that-apply (CATA) is a methodology for sensory product characterization that can be used by consumers. One of its main advantages is that the consumer can select several attributes for the same product instead of only focusing on one aspect [1,2]. The selection of words included in the CATA questionnaire could be descriptors used by trained panels or could be selected from previous focus groups or quantitative consumer studies [3,4].

However, one of the major paradigms of sensory analysis is that the attributes of a particular food are generally raised by a trained panel, and consumers are only asked how they perceive these attributes; that is, consumers do not have the opportunity to express how they perceive these characteristics in the first place. One problem related to this is that some of the attributes raised by the trained panel may not be relevant to consumers, or these attributes may be described by the panel differently from consumers would describe them [3,5].

In a focus group (FG), participants are more motivated to form new attitudes when interacting with other participants [6]. In addition, the participants’ natural vocabulary can be used to develop items for a questionnaire, or when there is a questionnaire that is already available and that will be applied in a population that is different from the one in which it was originally developed, the FG can be used to understand the meaning of the items to be explored [7].

In this study, the CATA test was applied to characterize three types of hot dogs, those with/without irradiation and salt reduction, and the FG technique was used to choose the CATA descriptors.

## 2. Materials and Methods

### 2.1. Hot Dog Preparation

The components mentioned in Table 1 were weighed separately and were homogenized in the following sequence in a bowl cutter (Trademark Tecmafrig, São Paulo, Brazil): the meat and salts were first chopped for 1 min. Then, the pork fat and half of the ice were added and processed for another 1 min. The remaining ice was added while cutting continued, followed by the addition of cassava starch, which was mixed until a homogeneous batter was obtained.

During the process, the temperature was maintained below 14 °C. The batter was stuffed into cellulosic casings (Viscofan do Brasil, São Paulo, Brazil) with a pneumatic filler (V25 Sirman) and were cooked in a smokehouse (SL 218 Arprotec, Valinhos, Brazil) at 60 °C for 30 min (dry air first, then steam after 15 min), then at 70 °C for 40 min, and finally at 80 °C until an internal temperature of 72 °C was reached. The hot dogs were cooled in running water before being stored in the refrigerator for 16 h. The casings were then removed, and the hot dogs were vacuum-packed and refrigerated at 4 °C.

The hot dogs were irradiated using a Cobalto-60 irradiator at the Nuclear Energy Research Institute (IPEN) in São Paulo, Brazil, at a rate of 5 kGy h-1 in static mode on the day after they were processed and packaged. The hot dogs with a reduced sodium content (1.25% NaCl) were given three different doses of irradiation: 1.5, 3.0, and 4.5 kGy, which were referred to as treatments F1.5, F3.0, and F4.5, respectively. Non-irradiated hot dogs with a reduced sodium content (F0) and non-irradiated hot dogs with a 2% sodium content were employed as control formulations (F2). For more information on the processing conditions and the impact of irradiation on hot dog sausages, read the previous paper written by Rodrigues et al. (2021) [8].

### 2.2. Participants and Study Design

The CATA and FG tests were performed in the Faculty of Animal Science and Food Engineering at the University of São Paulo, Pirassununga, Brazil. The tests were approved by the local Research Ethics Committee (no. 2.078.895).

An FG study with four groups of hot dog consumers was conducted. Each group was composed of eight participants. Participant selection was based on the criteria of being a regular consumer of hot dog sausages (at least once a week). To increase the CATA attributes through the FG, after the FG discussions, all of participants were served three hot dog samples, which were labeled as A, B, and C. These samples were selected so that there was a control sample with no sodium reduction and no irradiation (A = F2), a sample with sodium reduction without irradiation (B = F0), and a sample with sodium reduction and irradiated at the highest dose in the study (C = F4.5).

Each participant wrote five words (attributes) that best defined each sample in their opinion. Then, a discussion of the defined attributes was carried out to understand each meaning and to assemble different words with the same meaning. The most frequently mentioned attributes in all groups were selected to compose the list of descriptors for the CATA test. This methodology for collecting terms was selected in order to ensure that the attributes that were chosen to compose the CATA list are as well-recognized and common to consumers of this type of product as possible.

After selecting the attributes, the CATA test was applied to 119 hot dog consumers. The CATA test was performed at the Sensory Analysis Laboratory of the Faculty of Animal Science and Food Engineering at the University of São Paulo. Each consumer evaluated five different hot dog samples, namely: F0, F1.5, F3.0, F4.5, and F2. Samples were served in monadic form in randomized blocks.

### 2.3. Data Analysis

To evaluate the CATA results, a chi-square test was performed to determine if there was a link between the lines (treatments) and columns (attributes). The distribution of the samples in a plane was visualized through a graph generated by Principal Coordinate Analysis (PCoA) using the XLStat software (Version 2016, Addinsoft, Paris, France).

## 3. Results and Discussion

The attributes that were selected to compose the CATA list are arranged in Table 2. The terms were presented in a balanced way among the consumers in order to reduce the effect of position in the evaluation of the attributes.

**Figure 1:**
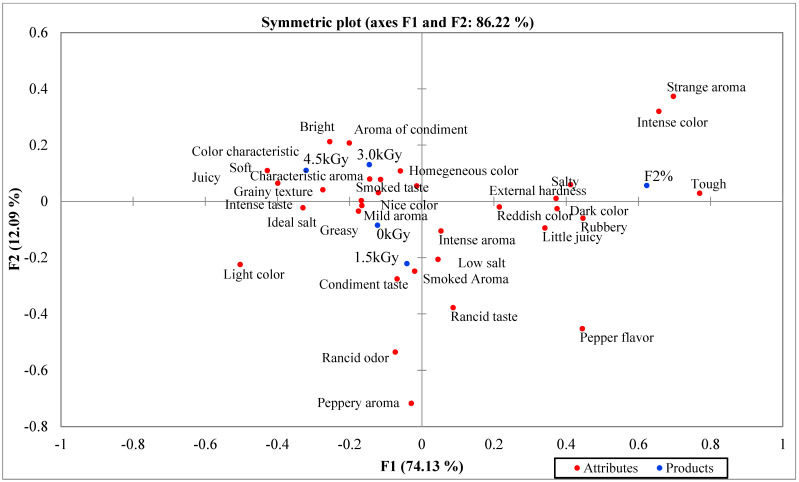
Graphic distribution of sodium-reduced and irradiated hot dog in relation to sensory attributes perceived by consumers in the CATA questionnaire. F2%—A (without sodium reduction and without irradiation. F0 kGy—B (with sodium reduction and without irradiation) (F0). F4.5 kGy—C (with sodium reduction and irradiated with the highest dose).

Figure 1 shows the results of the CATA test when evaluated by the consumers. The attributes for texture, “tough”, color, “intense color”, and the amount of salt, “salty”, were the most common descriptors associated with F2. For hot dog F0, characteristics such as “ideal salt”, “greasy”, and “mild aroma”, were assigned, while the F4.5 hot dog was described as “soft”, “grainy texture”, and “nice color”. The most differences across hot dog formulations were seen between those with and without salt reduction. The consumers only noticed minor effects from the irradiation process.

The sodium-reduced hot dog (F0) occupied a clearly distant position from the control sample without sodium reduction (F2). Hot dog F2 was described as being salty, tough, rubbery with external hardness, intense, and reddish in color. The hot dogs with a reduced sodium content remained close to each other. However, it is possible to detect some separation between the hot dogs that received the highest radiation doses (F3.0 and F4.5) from hot dogs F1.5 and F0. Hot dogs F3.0 and F4.5 were characterized as being homogeneous in color, having a smokey taste, an intense taste, soft texture, and characteristic aroma. Hot dog F0 was perceived as greasy and had mild aroma with an ideal amount of salt. Hot dog F1.5 was characterized as having a smokey aroma and condiment flavor.

These results indicate that sodium reduction had a strong impact on texture characteristics, while irradiation had some impact on the flavor of the products. The reduction in the sodium concentration may have impacted protein extraction during the comminution step when the hot dogs were being prepared. This may have impaired the formation of the protein network, leading to texture problems in the products. The impact of sodium reduction in meat products has been explored extensively by researchers, and the results include changes in texture, yield, flavor, and shelf life [9,10,11,12].

The irradiation of meat products can cause lipid oxidation, leading to the formation of a rancid taste and odor [13]. While these two attributes were not related to irradiated hot dogs, the consumers’ intensity perception of these attributes was not identified in relation to the samples.

The use of FG as method for selecting CATA descriptors for hot dogs was proven to be valid since the words listed in the CATA for these samples were attributes that typically characterize hot dog sausages [14,15].

## 4. Conclusions

The irradiated hot dogs had similar descriptors as the non-irradiated hot dogs in the CATA evaluation. However, sodium reduction caused important impacts on the sensory attributes of this product. This means that the application of radiation did not create major changes when compared to sodium reduction. The higher the dose of radiation applied, the greater the change in the flavor of the hot dogs. Meanwhile, F1.5 hot dogs were very close to F0.

The use of FG as a method to select CATA descriptors proved to be a valid attribute selection method that was capable of translating real consumer perceptions.

## Figures and Tables

**Table 1:** Formulations of irradiated and non-irradiated hot dogs.

Ingredients	Sodium Reduced	Control (F2)
Ground beef	60	60
Pork backfat	10	10
Cassava starch (Yoki, Brazil)	2	2
Salt (NaCl) (Cisne, Brazil)	1.25	2
Sodium Nitrite (Cori, Brazil)	0.015	0.015
Sodium erythorbate (Cori, Brazil)	0.05	0.05
Sodium tripolyphosphate (Cori, Brazil)	0.25	0.25
Sausage seasoning (New max, Brazil)	0.5	0.5
Carmine coloring	0.05	0.05
Water/Ice	25.89	25.14
Total	100	100

**Table 2:** Attributes selected to compile the CATA test list applied to hot dogs with sodium reduction and that had been irradiated.

Appearance	Aroma	Taste	Texture
Nice color	Strange aroma	Ideal salt	External hardness
Dark color	Smoked aroma	Little salt	Tough
Bright color	Intense aroma	Salty	Rubbery
Reddish	Mild aroma	Intense taste	Soft
Characteristic Color	Characteristic aroma	Rancid taste	Juicy
Intense color	Rancid odor	Condiment taste	Slightly succulent
Brightness	Condiment aroma	Pepper flavor	Grainy texture
Homogeneous color	Pepper aroma	Smoked flavor	Fatty
